# Spatial Distribution of Calcium-Gated Chloride Channels in Olfactory Cilia

**DOI:** 10.1371/journal.pone.0015676

**Published:** 2010-12-30

**Authors:** Donald A. French, Dorjsuren Badamdorj, Steven J. Kleene

**Affiliations:** 1 Department of Mathematical Sciences, University of Cincinnati, Cincinnati, Ohio, United States of America; 2 Department of Cancer and Cell Biology, University of Cincinnati, Cincinnati, Ohio, United States of America; Duke University, United States of America

## Abstract

**Background:**

In vertebrate olfactory receptor neurons, sensory cilia transduce odor stimuli into changes in neuronal membrane potential. The voltage changes are primarily caused by the sequential openings of two types of channel: a cyclic-nucleotide-gated (CNG) cationic channel and a calcium-gated chloride channel. In frog, the cilia are 25 to 200 µm in length, so the spatial distributions of the channels may be an important determinant of odor sensitivity.

**Principal Findings:**

To determine the spatial distribution of the chloride channels, we recorded from single cilia as calcium was allowed to diffuse down the length of the cilium and activate the channels. A computational model of this experiment allowed an estimate of the spatial distribution of the chloride channels. On average, the channels were concentrated in a narrow band centered at a distance of 29% of the ciliary length, measured from the base of the cilium. This matches the location of the CNG channels determined previously. This non-uniform distribution of transduction proteins is consistent with similar findings in other cilia.

**Conclusions:**

On average, the two types of olfactory transduction channel are concentrated in the same region of the cilium. This may contribute to the efficient detection of weak stimuli.

## Introduction

Cilia are key sensory organelles for the detection of light, chemical stimuli, motion, and temperature [1]–[4]. These stimuli are transduced by a variety of proteins in the ciliary membrane, including receptors, G-proteins, and channels. Opening and closing of the channels leads to a receptor current that changes the membrane potential of the cell following stimulation.

For various sensory cilia, the ratio of length to diameter ranges from about 10 to 700. This geometry is likely to influence the efficiency with which the cilium detects chemical stimuli. The cilium is often thought of as an antenna [1], [3] that extends the cellular surface area, increasing the probability that a stimulus molecule will collide with a sensory receptor. From this standpoint it would seem optimal to express the transduction proteins evenly along the length of the cilium. However, electrical signals may be significantly attenuated during conduction in fibers as long and thin as a cilium [5], [6]. During a strong response, channels positioned near the base of the cilium should change the cellular potential more effectively than channels near the distal tip. Another design consideration is the proximities among the several proteins that contribute to transduction. Whether a second messenger reaches its target depends on how far it can travel before being degraded or transported out of the cilium. In general, it is not yet known what spatial distribution of ciliary transduction proteins is optimal for sensory function.

In the olfactory cilia of vertebrates, a mechanism for electrochemical transduction of odor molecules is well documented (reviewed in [7], [8]). Through a G-protein-coupled mechanism, odor binding leads to the generation of cAMP within the cilium. cAMP gates channels in the ciliary membrane that allow a depolarizing influx of cations, including Ca^2+^. Ca^2+^ then gates Cl^−^ channels that cause a further depolarization via an efflux of Cl^−^. By modeling electrical recordings made in single olfactory cilia, we now infer that the Cl^−^ channels are expressed in a narrow band closer to the base of the cilium. This is the same location inferred for the cAMP-gated channels in our previous study [9].

## Materials and Methods

### Ethics statement

All experiments were approved by the University of Cincinnati's Institutional Animal Care and Use Committee (protocol 06-04-06-01) and conducted in accordance with the recommendations in the Guide for the Care and Use of Laboratory Animals of the National Institutes of Health.

### Ciliary patch procedure

Electrical recordings were made from olfactory cilia of Northern grass frogs (*Rana pipiens*) as described previously [10]. A frog olfactory epithelium was dissociated by mechanical shredding. One cilium of an isolated olfactory receptor neuron was drawn into a patch pipette, and a high-resistance seal was made where the olfactory knob meets the base of the cilium. The cilium was then excised from the cell, resulting in an inside-out patch configuration. The pipette containing the cilium was moved to a pseudointracellular bath so that the intracellular side of the cilium was exposed to the bath solution.

**Figure 1:**
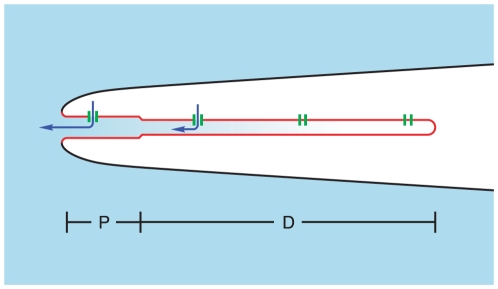
Schematic diagram of the experimental method. A single cilium (shown in red) from a frog ORN is sealed inside a glass micropipette (black). The base of the cilium, which is attached to the dendrite in vivo, is shown at the left. The distal tip is at the right. The base of the cilium is open and allows diffusion of Ca^2+^ (light blue) from the surrounding bath into the cilium. As Ca^2+^ reaches Cl^−^ channels (green) in the ciliary membrane, it gates them, allowing an inward current (efflux of Cl^−^, dark blue arrows). The uniform distribution of channels shown does not represent the experimental finding. At the bottom are shown the approximate extents of the proximal (P) and distal (D) segments of the cilium as described by Reese [11]. Only the relative diameters and the relative lengths of the proximal and distal segments are drawn to scale. Reprinted from [9], Flannery et al. (2006), copyright 2006, with permission from Elsevier.

The first pseudointracellular bath contained a low level of free Ca^2+^ (<0.1 µM). The leak current measured in this bath was subtracted from all subsequent measurements. The leak current at −50 mV averaged −15±1 pA (*n* = 301). The pipette containing the cilium was then transferred through the air to a bath containing a higher level of free Ca^2+^ (7 to 300 µM). Contact with the bath initiated the diffusion of the bath solution into the cilium (Fig. 1). The resulting Cl^−^ channel activation was recorded over a period from 4 to 20 s, depending on the cilium. Video of the patch procedure was recorded, and ciliary length was estimated by playing back the video recording one frame at a time. Multiple tests were conducted with many of the cilia. Between tests, the cilium was placed in the low-Ca^2+^ bath for about 1 min, which was longer than the time required for the current to return to the leak value.

The extracellular (pipette) solution contained (in mM): LiCl, 118; MgCl_2_, 2; CaCl_2_, 1; Li-HEPES, 5; pH 7.2. Compositions of the pseudointracellular solutions used are given in Table S1. In all solutions, Li^+^ was used instead of Na^+^ to prevent Ca^2+^ fluxes from sodium/calcium exchange [12], [13]. In some experiments, BAPTA was replaced with either of two Ca^2+^ buffers of lower affinity: 1,2-bis(2-amino-5-bromophenoxy)ethane-*N,N,N′,N′*-tetraacetic acid (dibromoBAPTA) or *N*-carboxymethyl-*N′*-(2-hydroxyethyl)-*N,N′*-ethylenediglycine (HEDTA). Apparent association constants *K′_Ca_* between Ca^2+^ and the buffers and were determined by Scatchard analysis [14] with a Ca^2+^-specific electrode (Orion 932000). *K′_Ca_* values were 6.3×10^6^ M^−1^ for BAPTA, 8.1×10^5^ M^−1^ for dibromoBAPTA, and 6.7×10^4^ M^−1^ for HEDTA. A Ca^2+^ buffer was included in every bath solution even though the buffer was saturated in some cases.

With all solutions used, the Ca^2+^-activated Cl^−^ current reversed near 0 mV. For electrical recording, both the recording pipette and chamber were coupled to an Axopatch 200B patch-clamp amplifier by Ag/AgCl electrodes. All recordings were done under voltage-clamp at room temperature (25°C). Current was sampled at 80 to 500 Hz by pCLAMP 5.7.1 software (Axon Instruments/Molecular Devices, Sunnyvale, CA).

### Synopsis of the computational modeling

To simulate the diffusion experiments, we used two computational procedures involving a model of the experiment. For each experimental recording of current vs. time, an inverse solution was used to generate a channel density function. The accuracy of the density function was then assessed by using this density function as input for a forward model. The forward model makes predictions about the time course of the current through the Cl^−^ channels, given a channel density function. The predicted current was compared to the experimental result. In the model, a distance of 0 represents the proximal end of the cilium (i.e. the end that is close to the basal body).

### Forward biophysical model

**Figure 2:**
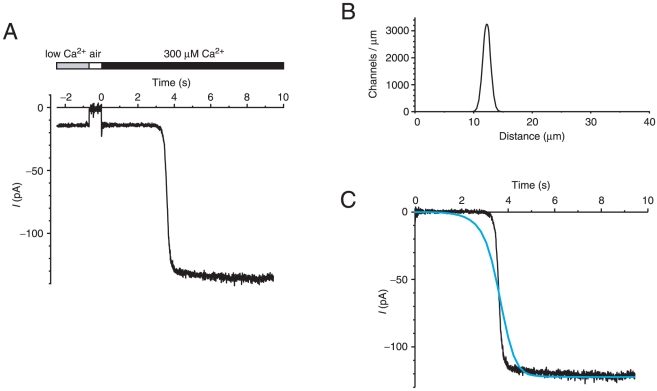
Time course of the Cl^−^ current activated by diffusion of Ca^2+^ into a cilium. (A) At the start of the recording, the 40-µm-long cilium was in a pseudointracellular bath containing <0.1 µM free Ca^2+^ and showed a leak current of −14 pA. The cilium was briefly moved through the air, during which time the current was 0 pA. At the time labeled 0, the cilium was immersed in a bath containing 300 µM free Ca^2+^. After a delay, this activated a current with a steep slope. The onset (time to reach 10% of the activated current) was 3.4 s. Both baths contained 2 mM BAPTA. Pipette potential was clamped at −50 mV throughout the experiment. (B) The channel density function predicted by the inverse solution applied to the recording shown in (A). The density function consists of a band of 5400 channels centered at a distance 12.2 µm from the base of the cilium. (C) A forward calculation from the density function shown in (B) predicted the current shown as a blue line. The original recording (A), with the leak current subtracted, is shown again in (C) for comparison.

A computational model was used to make predictions about channel currents resulting from diffusion of Ca^2+^ into a cilium, given a particular ion channel density function (e.g. Fig. 2). The model accounts for several physical processes, including diffusion of Ca^2+^, diffusion of the buffer, binding of Ca^2+^ to the buffer and to the Cl^−^ channels, channel activation, and cable-conduction effects. Diffusion and binding of Ca^2+^ were modeled by a nonlinear time-dependent partial differential equation that also depends on the channel distribution. The rapid buffer approximation was used to reduce the number of equations. Binding and activation of channels by Ca^2+^ were both represented by a single two-parameter Hill equation. The membrane potential satisfied a second-order boundary value problem that depends on the channel distribution, the concentration of Ca^2+^, and time. These equations were approximated by basic finite difference schemes. A detailed description of the model is given in Text S1.

Some minor factors were not included in the model. It does not account for a small leak conductance in the ciliary membrane [15]. Only cilia with minimal leak conductance (input resistance ≥1 GΩ) were used. No pathways for conducting Ca^2+^ across the membrane were modeled. Sodium/calcium exchange was not modeled, since the exchange was prevented as described above. Since no ATP was provided, Ca^2+^-ATPase was assumed to be inactive. The diameter of the cilium decreases abruptly from 0.28 µm to 0.19 µm where the proximal and distal segments meet [16] (see also Fig. 1). Because modeling an abrupt decrease in diameter is difficult [17], [18], our model assumed that the cilium is a cylinder of constant diameter (0.28 µm). It was assumed that unhindered diffusion of Ca^2+^ is possible in the entire volume of the cilium. Binding of Ca^2+^ to the Cl^−^ channels was considered to be much faster than diffusion. Other possible membrane-associated Ca^2+^-binding sites were not modeled because quantitative data are unavailable. Finally, capacitative current was ignored. We estimate that the time constant of a cilium should be ∼3 ms, which is much faster than the events recorded here.

### Inverse solution

The primary unknown in our biophysical model is the spatial distribution of the Cl^−^ channels, and it was the aim of our modeling and experiments to generate a function quantifying this unknown. The inverse solution offers a systematic way of generating density functions, using the measured time-dependent activation of Cl^−^ channels as input. The inverse solution has the same components as those described in the forward biophysical model. Through a series of iterations, the inverse solution makes an approximation of the channel density function *ρ(x)*, where *x* represents distance along the length of the cilium. The iterative process is described in greater detail in Text S1.

The noisy raw current data were smoothed by a moving average with 7 points and then used to generate an inverse solution (a channel density function *ρ(x)*). Previous studies [9], [19], [20] suggested that narrow Gaussian channel distributions provide current profiles that fit the experimental currents well. The inverse solver performs an initial search for the location of the channels along the length of the cilium using the asymptotic formulas from Badamdorj et al. [20]. It then uses the minimization function of MATLAB (MathWorks, Natick, MA) to further refine the channel distribution.

### Data selection and analysis

The forward model should convert the inverse solution (a density function) to a current record the same as the experimental recording in every respect except noise. A given channel density function was rejected when the measured and predicted currents did not match. The differences between the recording and the current predicted by the model were quantitated as the relative least squares error (see Text S1). If this exceeded 0.2, we rejected the solution. Of 301 recordings analyzed, 239 (79%) of the density functions were judged to be credible and the remaining 62 (21%) were rejected.

Onset time was defined as the time it took for the first 10% of the Ca^2+^-activated current to appear. Results of repeated experiments are reported as mean ± SEM.

## Results

When a cilium was quickly lowered from the air into a pseudointracellular bath containing 300 µM free Ca^2+^, the membrane current was initially stable. Following a delay, the current increased rapidly (Fig. 2A). The delayed current was due to gating of Cl^−^ channels in the ciliary membrane [21] by Ca^2+^ that diffused from the bath into the cilium. In the example shown in Fig. 2A, the onset of the Ca^2+^-induced current (time to reach 10% of the maximal current) occurred 3.4 s after the cilium was placed in the Ca^2+^-containing bath. In excised olfactory sensory endings from rat [22] or mouse [23], the Ca^2+^-activated Cl^−^ current is gradually reduced by rundown and desensitization to Ca^2+^. However, the analogous current in frog is stable for at least 10 min [21]. For a given cilium, the onset time and the amplitude of the Ca^2+^-activated current varied little over the duration of a typical experiment (Fig. S1). In all recordings made in a standard bath (2 mM BAPTA, 300 µM free Ca^2+^), the onset time averaged 3.57±0.20 s (154 recordings from 61 cilia, range 0.51 to 14.6 s). There was a positive correlation between the onset time and the length of the cilium (Fig. S2).

**Figure 3:**
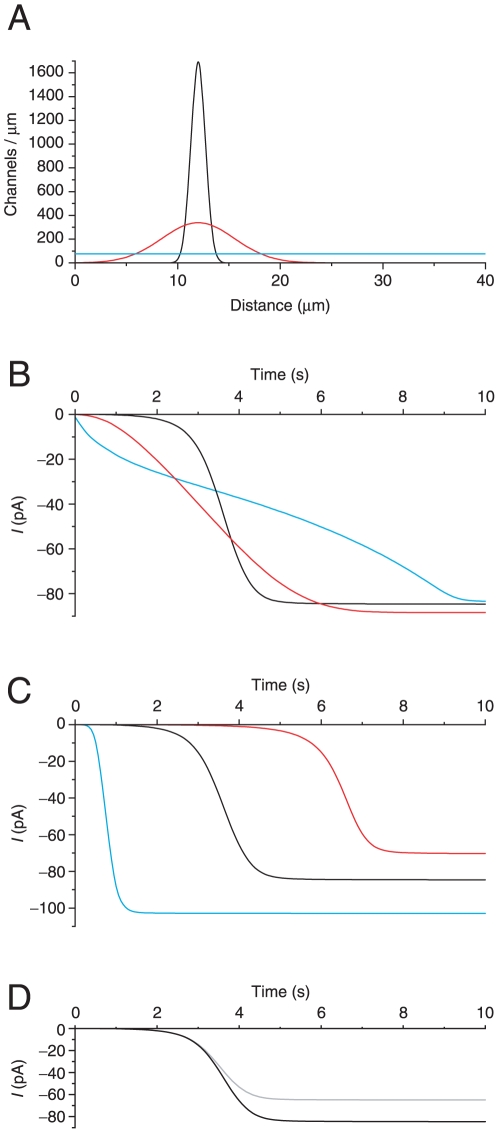
Currents predicted by the forward model for various assumed channel distributions. A hypothetical cilium was assumed to be 40 µm long with 3000 Ca^2+^-gated Cl^−^ channels distributed along its length. The forward model was used to predict the current as a function of time assuming the cilium was placed in a bath containing 2 mM BAPTA and 300 µM free Ca^2+^ with the pipette potential clamped at −50 mV. (A) Three assumed channel distributions are shown. The blue line shows a uniform distribution. The others show the channels distributed as Gaussian bands. The widths of the bands at points where the height is 10% of the peak height are 3 µm (black) and 15 µm (red). (These correspond to *δ_w_* = 1 and *δ_w_* = 5 using the terminology defined in Text S1.) Each of these two bands was centered at a distance of 12 µm from the base of the cilium. (B) The predicted currents assuming the channel distributions shown in (A) (using corresponding colors). The distribution shown in black most closely represents the experimental results (e.g. Fig. 2A). (C) Currents were predicted assuming a Gaussian distribution of channels of width 3 µm centered at each of three distances from the base of the cilium: 5 µm (blue), 12 µm (black), or 20 µm (red). Current amplitudes at infinite time were −103 pA (blue), −85 pA (black), and −70 pA (red). In the absence of cable-conduction loss, the 3000 channels would produce a total current of −113 pA at −50 mV. The smaller plateau values were due to this loss, which is greater as the channels are placed near the distal tip. The onset times (times to reach 10% of the plateau current) were 0.5 s (blue), 2.7 s (black), and 5.5 s (red). In all of the examples above, the ciliary diameter was assumed to be 0.28 µm. (D) Currents were predicted assuming a ciliary diameter of 0.28 µm (black) or 0.19 µm (gray) and a narrow band of 3000 channels centered at 12 µm. Assuming the smaller diameter changed the onset time from 2.7 s to 2.6 s, while the predicted current plateau was reduced from −85 pA to −65 pA. The three black curves shown in (B), (C), and (D) are identical.

Our goal was to infer the spatial distribution of the Cl^−^ channels from the time course of the experimental current. To that end, a forward model of the experiment was first developed. The model predicts that the rate at which the current appears depends strongly on the spatial distribution of the channels (Fig. 3). Modeling a uniform channel distribution (Fig. 3A,B, blue) or a broad band of channels (Fig. 3A,B, red) resulted in a rate of current development much slower than was measured in the experiments. Only a model that assumed a narrow band of channels (Fig. 3A,B, black) approximated the experimentally observed rate of current. For that reason, subsequent models assumed that the channel distribution could be well represented as a narrow band. For a given narrow band of channels, the onset of the modeled current depended strongly on the position of that band along the cilium's length (Fig. 3C). Given that strong dependence, the onset of the experimental current could be used to infer the location of the band of Cl^−^ channels. Reducing the modeled diameter of the cilium had little effect on the predicted onset time but reduced the predicted plateau current (Fig. 3D, gray). In a cilium of smaller diameter, cable-conduction loss is greater, accounting for the smaller plateau current.

**Figure 4:**
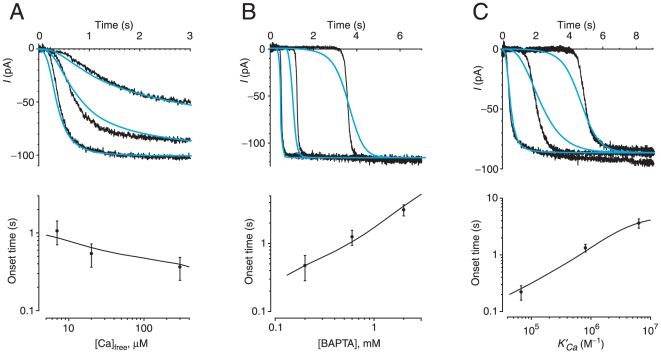
Effects of Ca^2+^-buffering parameters on the observed and predicted Cl^−^ currents. (A) The upper figure shows recordings made after a single 30-µm-long cilium was placed in a bath containing 300, 20, or 7 µM free Ca^2+^. All solutions contained 2 mM HEDTA, and pipette potential was clamped at −50 mV. The onset times were 241 ms (300 µM free Ca^2+^), 367 ms (20 µM), and 564 ms (7 µM). The three blue curves are the currents predicted by the forward model assuming that 3800 Cl^−^ channels were present in a narrow band centered 11.5 µm from the base of the cilium. In the lower figure, the points show the experimental relation between [Ca^2+^]_free_ and onset time (means determined in 4 cilia). The line shows the relation predicted by the forward model for a 60-µm-long cilium with a band of channels centered 17.3 µm from the base. (B) The upper figure shows recordings made after a single 40-µm-long cilium was placed in a bath containing 0.2, 0.6, or 2 mM BAPTA. All solutions contained 300 µM free Ca^2+^, and pipette potential was clamped at −50 mV. The onset times were 450 ms (0.2 mM BAPTA), 1.17 s (0.6 mM), and 3.39 s (2 mM). The three blue curves are the currents predicted by the forward model assuming that 4960 Cl^−^ channels were present in a narrow band centered 12.2 µm from the base of the cilium. In the lower figure, the points show the experimental relation between [BAPTA] and onset time (means determined in 8 to 12 cilia). The line shows the relation predicted by the forward model for a 50-µm-long cilium with a band of channels centered 14 µm from the base. (C) The upper figure shows recordings made after a single 68-µm-long cilium was placed in a bath buffered with HEDTA, dibromoBAPTA, or BAPTA. In all solutions, [Ca^2+^]_free_ was 300 µM and [buffer] was 2 mM. Pipette potential was clamped at −40 mV. The onset times were 255 ms (HEDTA), 1.48 s (dibromoBAPTA), and 4.50 s (BAPTA). The three blue curves are the currents predicted by the forward model assuming that 4720 Cl^−^ channels were present in a narrow band centered 13.5 µm from the base of the cilium. In the lower figure, the points show the experimental relation between the buffer's apparent association constant for Ca^2+^ (*K′_Ca_*) and onset time (means determined in 13 to 21 cilia). The line shows the relation predicted by the forward model for a 50-µm-long cilium with a band of channels centered 13.8 µm from the base. From each recording shown in the upper figures, a small leak current (−9 to −15 pA) has been subtracted.

We verified that the model accurately represents buffering of Ca^2+^ by the exogenous buffers supplied (Fig. 4). When a given cilium was tested in baths with different concentrations of free Ca^2+^, the time course of the Cl^−^ current was found to depend strongly on [Ca^2+^]_free_ (Fig. 4A). The onset of the current was faster when [Ca^2+^]_free_ in the bath was higher. It was possible to define a band of channels such that the forward model successfully predicted the experimental currents measured in each of three baths with different [Ca^2+^]_free_ (Fig. 4A, top, blue curves). The relation between [Ca^2+^]_free_ and onset time measured in a population of cilia was also well modeled (Fig. 4A, bottom). As expected, the time course of the Cl^−^ current was also sensitive to changes in the concentration of buffer (Fig. 4B, top) and the apparent association constant *K′_Ca_* between the buffer and Ca^2+^ (Fig. 4C, top). These effects were also well modeled (Fig. 4B,C). The model was less successful in representing the rate at which the Cl^−^ current appeared following the initial onset. In most but not all experiments, the measured rate was faster than predicted by the model. There was a moderate positive correlation between onset time and the relative least squares error of the fit (*r* = 0.18, *n* = 239, *P*<0.01). In other words, the model was somewhat more successful at fitting currents with faster onset times. The reason for this is unknown.

The model assumes that diffusion of Ca^2+^ is not biased by the applied voltage gradient. This was verified experimentally (Fig. S3). In each of 3 cilia, the onset times of the current were measured at two pipette potentials (+40 and −40 mV). The ratios of the onset times at the two voltages were 0.99, 0.99, and 1.02. The model also does not account for a second possible source of cytoplasmic Ca^2+^. Even in the absence of a gating ligand, the ciliary cyclic-nucleotide-gated (CNG) channels allow a small influx of Ca^2+^ [22], [24]. To test for an effect of this Ca^2+^ influx, the onset of the Cl^−^ current was measured in the presence and absence of 1 mM amiloride, which blocks the CNG channels [25], [26]. If a membrane Ca^2+^ influx were significant, it would decrease the onset time, while blocking the influx with amiloride would increase it. In 9 cilia tested, the onset time increased in the presence of amiloride by a factor of 1.07±0.03. This effect was not significant at the 95% confidence level. There was also no significant effect of amiloride on the maximum slope of the relation between current and time.

Using the computational model of the experiment, an inverse solution was developed (as detailed in Text S1). The inverse solution, given an experimental recording, finds a spatial channel distribution that is consistent with the recording. When applied to the recording shown in Fig. 2A, the inverse solution suggested a band of Cl^−^ channels centered at a distance of 12.2 µm from the base of the cilium (Fig. 2B). As a check, the forward model was used to predict the experimental current given this distribution of channels. The time course of the predicted current matched the experimental current well (Fig. 2C).

**Figure 5:**
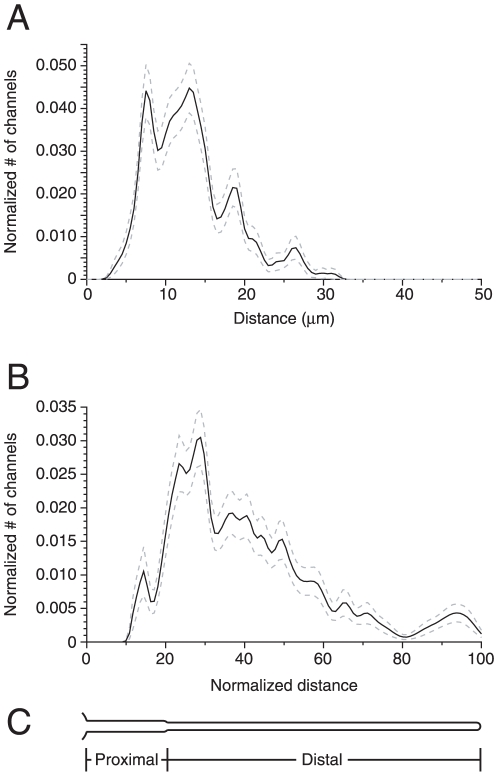
Average channel density function. (A) By inverse solution, channel density functions (e.g. Fig. 2B) were generated from each of 239 recordings from 59 different cilia. The black line shows the average density function; the dashed gray lines represent the standard errors. Recordings made under all of the Ca^2+^-buffering conditions described for Fig. 4 were included in the average. Each channel density function consisted of 111 points evenly spaced along the length of the cilium. Before averaging, each function was scaled to a total integrated area of 1.0. Thus the *y*-coordinate represents the fraction of the total channels in each of the 111 distance bins. Ciliary length averaged 36±1 µm (range 18 to 78 µm). On average, the inverse solutions reported 4040±260 channels per cilium (range 710 to 27930 channels per cilium). The peak of the inferred Gaussian channel distribution was on average 13.1±0.3 µm from the base of the cilium (range 3.6 to 31.2 µm), and the width of the peak was 2.8±0.01 µm (range 2.3 to 3.2 µm). The width was determined at the points where the height of the density function was 10% of the peak height. (B) As in (A), but each channel density function was also scaled to an arbitrary length of 100. (C) Schematic of an olfactory cilium showing the proximal and distal segments as described by Reese [11], plus a piece of the dendritic knob at the far left. The relative diameters are drawn to scale. The length is scaled to match the distance axis in (B).

When the inferred channel distributions from all 239 successfully modeled experiments were averaged, most of the Cl^−^ channels were found at distances of 5 to 15 µm from the base of the cilium (Fig. 5A). The average channel density function was also generated after normalizing the length of each cilium to an arbitrary value of 100 (Fig. 5B). In this case the distance from the base of the cilium to the peak of the density function was 29% of the length of the cilium.

**Figure 6:**
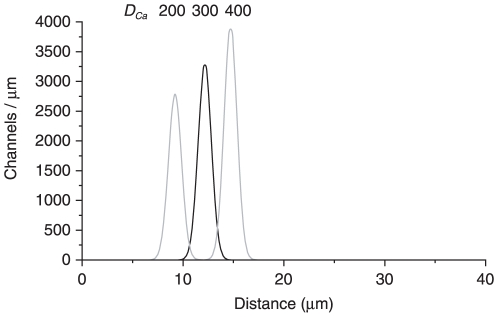
Sensitivity analysis of the model. For each of five parameters used in the model (unitary conductance of the channel *g_Cl_*, the Hill constant *n*, *K_1/2_* for activation of channels by Ca^2+^, the dissociation constant *K_B_* between Ca^2+^ and BAPTA, and the number of Ca^2+^-binding sites per channel *B_S_*), three values were assumed. For the first four parameters, the values were the mean experimental value and the mean plus or minus the standard error as shown in Table S2 of Text S1. For the number of binding sites, values of 1, 2, and 3 were assumed. For each of the resulting 3^5^ = 243 permutations of values, the inverse solver generated a channel density function. The average of the 243 functions is shown as a black curve. This average function peaked at 12.2 µm and represented a total (integrated area) of 5329 channels. For this analysis a sixth parameter, the diffusion coefficient of Ca^2+^ in the cilium (*D_Ca_*), was assumed to be 300 µm^2^ s^−1^. Repeating the analysis with *D_Ca_* = 200 µm^2^ s^−1^ gave an average density function centered at 9.2 µm with 4490 channels; assuming *D_Ca_* = 400 µm^2^ s^−1^ gave a function centered at 14.7 µm with 6230 channels (curves shown in gray). Varying *D_B_*, the diffusion coefficient of BAPTA, between 65 and 125 µm^2^ s^−1^ had no significant effect (not shown). The recording used for sensitivity analysis was the same as that shown in Fig. 2.

The model assumes constant values for the many parameters used (listed in Table S2 of Text S1). However, many of these values are associated with experimental errors. To determine how reasonable errors in some of these values affect the predictions of the model, we applied sensitivity analysis. For each of five parameters, three values were assumed as described in the legend of Fig. 6. For each of the resulting 243 permutations, the inverse solver identified a channel density function. There was very little variability among the inferred channel distributions; the average of all 243 functions is shown in Fig. 6 (black curve). Assuming different values for a sixth parameter, the diffusion coefficient of Ca^2+^ (*D_Ca_*), did modestly influence the position of the band of channels (Fig. 6, gray curves).

## Discussion

When a frog olfactory cilium is excised from the cell and immersed in a bath containing Ca^2+^, the Ca^2+^ diffuses down the length of the cilium. As free Ca^2+^ exceeds ∼2 µM at any point along the cilium, it gates some of the ciliary Cl^−^ channels present there [21]. In theory, the time course of activation of the Cl^−^ current depends on several factors, including the diffusion of Ca^2+^ and any mobile Ca^2+^ buffers down the cilium, the association of Ca^2+^ with any fixed or mobile Ca^2+^ buffers, and the spatial distribution of the Ca^2+^-gated Cl^−^ channels along the cilium. The onset of the Cl^−^ current should become faster as any endogenous mobile buffers leave the cilium. In fact, the onset was stable for several minutes following excision of the cilium (Fig. S1). It is likely that any endogenous mobile Ca^2+^ buffers diffuse out of the cilium quickly following excision. Because the Ca^2+^-gated Cl^−^ channels are present at high density in the cilium (62 to 78 channels µm^−1^, averaged over the length of the cilium [22], [27]), we assumed that they are the major fixed Ca^2+^ buffer. Constants governing the diffusion of Ca^2+^ and its interaction with the added buffer and with the Cl^−^ channels are available (see Table S2 of Text S1). However, the spatial distribution of the Cl^−^ channels in a given cilium was unknown.

To infer the channel distribution, we created a computational model of the experiment. A forward model, given the experimental variables, predicted the time course of the Cl^−^ current after immersion of the cilium in a bath containing Ca^2+^. When compared with experimental findings, the model successfully represented the effects of changes in [Ca^2+^]_free_, the concentration of added Ca^2+^ buffer, and the apparent association constant *K′_Ca_* between the buffer and Ca^2+^ (Fig. 4). The forward model also indicated that the time course of the current strongly depends on the spatial distribution of the Cl^−^ channels (Fig. 3). In principle, then, the time course of the experimental current is a sensitive indicator of the channel distribution.

With few exceptions, immersion of a cilium into a bath containing 300 µM free Ca^2+^ produced a current that developed in three phases: an initial delay, a rapidly developing current, and a stable plateau. The initial delay typically lasted several seconds when the bath contained a high-affinity Ca^2+^ buffer (Fig. 4B,C). One could imagine that this delay in part reflects solution mixing after lowering the cilium into the bath. However, the onset time (time to reach 10% of the current) was in several cases <10 ms when the lower-affinity buffer HEDTA was used. In general, the forward model correctly predicted delayed onsets when high-affinity Ca^2+^ buffers were used (Fig. 4B,C). Theoretically, the delay should also be influenced by fixed Ca^2+^ buffers, particularly in the proximal part of the cilium. The distributions and binding constants of such buffers are unknown and were not included in the model. The Ca^2+^-activated Cl^−^ channel itself is one fixed Ca^2+^ buffer. We found that varying the number of Ca^2+^-binding sites per channel had little effect on the predicted current (Fig. 6), even when a uniform spatial distribution of the channels was modeled (not shown).

Following the initial delay, the rate of appearance of the Cl^−^ current was strikingly fast (Figs. 2A, 4B,C, S1, S3). With few exceptions, this rate was underestimated by the forward model. (This was also true in a previous study of the CNG channels [9].) The rate was strongly dependent on the width of the modeled spatial distribution of channels (Fig. 3B). Only a very narrow band of channels predicted a rate of current that approached the experimental results. The experimental rate could be better matched by assuming higher values for the diffusion coefficient of Ca^2+^, the affinity of the buffer for Ca^2+^, or the Hill constant for channel activation. However, there is no independent evidence to justify such assumptions. The rate of current should also be increased if [Ca^2+^]_free_ near the Cl^−^ channels is greater than the low level (<0.1 µM) present in the buffered solution initially used to fill the cilium. The ciliary CNG channels allow a small Ca^2+^ influx even in the absence of cAMP [22], [24], and it was thought that this additional Ca^2+^ source might account for the rapid rate of Cl^−^ current. However, blocking the CNG channels with amiloride had no significant effect on the rate. It is possible that the rate of current is influenced by the small, abrupt decrease in diameter just proximal to the inferred band of channels (Fig. 5). However, we did not model the change in diameter and cannot predict with confidence how it might affect the rate. The final phase of each recording, a stable current, is reached as the cilium becomes filled with the high-Ca^2+^ solution. This represents the macroscopic current expected from all of the Cl^−^ channels, given the particular level of free Ca^2+^ in the bath. The current is reduced by cable-conduction loss in the cilium (see Text S1).

Using the computational model of the experiment, an inverse solver was developed. Given the experimental record of Cl^−^ current as a function of time, the inverse solver identified a spatial distribution of channels consistent with the model and the recording. Since only a narrow band of channels could account for the rapid phase of the current, the inverse solver was designed within that constraint. The position of the peak of channel density varied among the 60 cilia studied. In no case was a peak identified in the 7% of the cilium closest to the base. Most often the peak was at a point with a distance from the base equal to 20% to 50% of the ciliary length (Fig. 5B). It was less common to find the channels in the half of the cilium that includes the distal tip. On morphological grounds, the cilium can be divided into proximal and distal segments (Fig. 5C; [11]). The proximal segment, which includes about 20% of the length of the cilium in frog [11], has a larger diameter (0.28 µm) and a complete axoneme with a (9×2) + 2 arrangement of microtubules [16], [28]. The distal segment is 0.19 µm in diameter and has singlet outer microtubules. The peak of the Cl^−^-channel distribution was usually found in the proximal half of the distal segment. The inferred peak position has a modest dependence on the assumed diffusion coefficient of Ca^2+^ (*D_Ca_*), which was modeled over the range of 100 to 300 µm^2^ s^−1^ (Fig. 6). In a model of the rat olfactory cilium, *D_Ca_* was inferred to be as low as 89.5 µm^2^ s^−1^ [22]. However, that model assumed a uniform spatial distribution of the CNG and Cl^−^ channels.

The distribution of Cl^−^ channels has been estimated by another method that is independent and more direct [29]. Intact olfactory receptor neurons of the newt were dialyzed with caged Ca^2+^, which diffused into the cilia. Focal laser irradiation of a cilium released Ca^2+^, and the resulting current was used to estimate the density of Cl^−^ channels near the site of irradiation. In most of 8 cilia examined, channel density declined gradually with distance from the base of the cilium. A similar trend was seen in our *average* channel distribution (Fig. 5). However, our individual recordings were best modeled by assuming that most of the channels were within a band 2 to 3 µm in width (Figs. 2B, 3A). The work of Takeuchi et al. [29] did not reveal such sharp distributions. However, comparisons are complicated by differences in the ciliary lengths. The frog cilia we used ranged from 18 to 78 µm in length. The newt cilia were much shorter (ranging from 4 to 12 µm), and the lengths studied by irradiation ranged from 2 to 9 µm (Fig. 10H of Takeuchi & Kurahashi [29]).

It is worth considering how many Cl^−^ channels can be accommodated by a short length of the cilium. The results suggest that most of the 4040 channels in an average cilium were confined to a 2.8-µm length of the cilium. This corresponds to a density of 2420 channels µm^−2^, assuming the distal ciliary diameter of 0.19 µm [16], [28]. If each Cl^−^ channel has a diameter of 10 nm (as estimated for the CNG channel [30]), then the geometric limit of channel density would be 11500 channels µm^−2^, assuming hexagonal close packing. Thus the predicted channel density is high but not out of the realm of possibility.

Olfactory cilia increase the neuronal surface area by a factor of about 40 [31]. One might guess that a uniform distribution of transduction proteins along the entire length of the cilium would provide optimal function. Consistent with this idea, odor receptors have been detected by immunocytochemistry along much of the ciliary length [32], [33]. However, it is becoming apparent that the transduction proteins of sensory cilia are often restricted to discrete zones. A voltage-gated Ca^2+^ channel that controls flagellar motion in *Chlamydomonas* is confined to the distal region of the flagellum [34]; these flagella are structurally similar to the cilia of vertebrates. In *Drosophila*, two TRP channels that support ciliary mechanotransduction are segregated within two distinct zones, one distal and one proximal [35]. In motile cilia of human airway epithelium, some bitter receptors are expressed in proximal bands and others more distally [36]. In olfactory cilia too, evidence has long suggested that transduction capacity is not uniformly distributed along the length. From measurements of the latencies of odor responses, Getchell et al. [37] inferred that only the proximal portions of the cilia actively contribute to transduction. Similarly, Adamek et al. [38] found in frog that most of the odor-induced epithelial potential was contributed by the 40 µm of the cilium closest to the dendrite. Ultrastructural studies showed that several proteins involved in odor transduction (the CNGA2 channel subunit, G_olfα_, and the type III adenylyl cyclase) are preferentially expressed in the distal segment of the cilium [39], [40].

Our own studies confirm that the functional CNG [9] and Cl^−^ channels (Figs. 2,5) that serve odor transduction do not have uniform spatial distributions. The channel densities are highest in the proximal half of the distal segment of the cilium (Fig. 5). Channels are rare toward either end of the cilium. It is surprising that few channels reside near the base of the cilium, where proximity to the dendrite could result in larger currents not reduced by cable-conduction loss. An explanation suggested by Quarmby [41] for *Chlamydomonas* may apply here as well. In *Chlamydomonas*, intraflagellar free Ca^2+^ concentrations above 1 µM cause the flagellum to detach near its base [42], [43]. During odor transduction in vertebrates, Ca^2+^ influx through the CNG channels routinely elevates intraciliary free Ca^2+^ above 1 µM [7], [44]. Were CNG channels expressed near the base of the cilium, the resulting elevation of Ca^2+^ might lead to ciliary detachment.

During odor transduction, the Ca^2+^-gated Cl^−^ channels work in concert with the CNG channels. Odor binding leads to production of cAMP, which gates the CNG channels. Ca^2+^ entering through the CNG channels then activates the Cl^−^ channels, greatly amplifying the depolarization (reviewed in 7,8,44). This amplification should be most efficient if the Cl^−^ channels are located near the CNG channels, particularly given that diffusion of cAMP and Ca^2+^ is restricted along the length of the cilium [45]. The spatial distributions of both channels have been estimated by the methods described here. The distributions averaged across many cilia are remarkably similar (Fig. 5; Fig. 5 of [9]), with peaks at 28% (CNG channels) or 29% (Cl^−^ channels) of the ciliary length. Both averaged distributions show few channels in two regions: the distal half of the cilium and a segment near the base. In individual cilia, both the Cl^−^ and CNG channels were best modeled by a narrow band of channels. The results suggest that the two channel types are clustered together in a common region of the cilium. On a finer scale, one can further imagine that each Cl^−^ channel might be immediately adjacent to a CNG channel. However, the only evidence to date suggests that the channels distribute randomly within their zones of expression [22].

## Supporting Information

Table S1 **Compositions of pseudointracellular solutions.** [Ca^2+^]_free_ in solutions with Ca^2+^ buffers was estimated as described previously [21]. The measured apparent association constants *K′_Ca_* between Ca^2+^ and the buffers are given in Materials and Methods. In addition, each solution contained (in mM): LiCl, 115; MgCl_2_, 2; Li-HEPES, 5; pH 7.2. The first solution shown (low-Ca^2+^ solution, [Ca^2+^]_free_ <0.1 µM) was used at the start of each experiment and between tests with solutions containing higher [Ca^2+^]_free_. For this solution, the value of [Ca^2+^]_total_ shown represents Ca^2+^ present in the distilled water used. In solutions with 300 µM free Ca^2+^, the Ca^2+^ buffer was saturated.
(PDF)

Figure S1 **Stability of the ciliary Cl^−^ current.** (A) A pipette containing a 24-µm-long cilium was placed in a bath containing 300 µM free Ca^2+^. Recordings in this second bath over a period of 9 min are shown. Between recordings the pipette and cilium were returned to the low-Ca^2+^ bath. (B) Across the 9 recordings, the onset time ranged from 1.80 to 2.06 s, and the final current ranged from −136 to −143 pA. Both baths contained 2 mM BAPTA. Pipette potential was clamped at −50 mV.
(EPS)

Figure S2 **Current onset time is greater in longer cilia.** The onset time of the Ca^2+^-activated Cl^−^ current was measured in each of 115 recordings from 49 different cilia. All recordings were made in a bath containing 300 µM free Ca^2+^ and 2 mM BAPTA. The pipette potential was clamped at −40 or −50 mV. The line represents the linear least-squares fit (*r* = 0.44, *P*<0.001).
(EPS)

Figure S3 **Time course of the Cl^−^ current is insensitive to voltage.** A pipette containing a 42-µm-long cilium was placed in a bath containing 300 µM free Ca^2+^ and 2 mM BAPTA. The time course of the Ca^2+^-activated Cl^−^ current was measured with the pipette potential clamped at −40 mV (lower recording). The cilium was placed in a low-Ca^2+^ bath for 1 min. Finally it was returned to the bath with 300 µM free Ca^2+^ but with pipette potential clamped at +40 mV (upper recording). Onset times of the current were 2.80 s at −40 mV and 2.86 s at +40 mV.
(EPS)

Text S1 **Details of the computational model.** The mathematical model consisting of partial differential equations that simulate the experiments involving the diffusion of Ca^2+^ into a cilium is described in this supplement. This model builds on earlier studies of both the Ca^2+^-gated Cl^−^ and CNG ion channels. The scientific computation procedure that is used to identify the Ca^2+^-gated Cl^−^ ion channel distributions is discussed.
(PDF)
