# Supplementary material for: Spatial Distribution of Calcium-Gated Chloride Channels in Olfactory Cilia
Source: PLoS One. 2010 Dec 30;5(12):e15676. doi: 10.1371/journal.pone.0015676 (PMC3012700; doi:10.1371/journal.pone.0015676)
Supplement: Table S1 — Compositions of pseudointracellular solutions. [Ca2+]free in solutions with Ca2+ buffers was estimated as described previously [21]. The measured apparent association constants K′Ca between Ca2+ and the buffers are given in Materials and Methods. In addition, each solution contained (in mM): LiCl, 115; MgCl2, 2; Li-HEPES, 5; pH 7.2. The first solution shown (low-Ca2+ solution, [Ca2+]free <0.1 µM) was used at the start of each experiment and between tests with solutions containing higher [Ca2+]free. For this solution, the value of [Ca2+]total shown represents Ca2+ present in the distilled water used. In solutions with 300 µM free Ca2+, the Ca2+ buffer was saturated. (PDF) [file pone.0015676.s001.pdf]

Table S1: Compositions of pseudointracellular solutions

buffer	$[buffer]_{total}$ (mM)	$[Ca^{2+}]_{total}$ (mM)	$[Ca^{2+}]_{free}$ (μM)	Figures
BAPTA	2.0	<0.01	<0.1	–
BAPTA	0.2	0.50	300	4B
BAPTA	0.6	0.90	300	4B
BAPTA	2.0	2.30	300	2, 4B, 4C, S1, S2, S3
dibromoBAPTA	2.0	2.30	300	4C
HEDTA	2.0	0.65	7	4A
HEDTA	2.0	1.17	20	4A
HEDTA	2.0	2.21	300	4A, 4C
