# Supplementary material for: Spatial Distribution of Calcium-Gated Chloride Channels in Olfactory Cilia
Source: PLoS One. 2010 Dec 30;5(12):e15676. doi: 10.1371/journal.pone.0015676 (PMC3012700; doi:10.1371/journal.pone.0015676)
Supplement: Text S1 — Details of the computational model. The mathematical model consisting of partial differential equations that simulate the experiments involving the diffusion of Ca2+ into a cilium is described in this supplement. This model builds on earlier studies of both the Ca2+-gated Cl− and CNG ion channels. The scientific computation procedure that is used to identify the Ca2+-gated Cl− ion channel distributions is discussed. (PDF) [file pone.0015676.s005.pdf]

Text S1: details of the computational model

Abstract

The mathematical model consisting of partial differential equations that simulate the experiments involving the diffusion of Ca^{2+} into a cilium is described in this supplement. This model builds on earlier studies of both the Ca^{2+}-gated Cl^- and CNG ion channels. The scientific computation procedure that is used to identify the Ca^{2+}-gated Cl^- ion channel distributions is discussed.

Introduction

We model the behavior of cytoplasmic Ca^{2+}, buffer and membrane potential in an olfactory cilium during experiments that involve the Ca^{2+}-gated Cl^- ion channels. The primary goal of this work is to elicit information on the distribution of the Ca^{2+}-gated Cl^- ion channels.

Our main goal in this supplement is to restate the mathematical model which was introduced in Badamdorj [46] and Badamdorj et al. [20] as well as the rapid buffer simplification.

Mathematical Model

Our model consists of equations for the membrane potential $v = v(x,t)$ and calcium concentration $c = c(x,t)$ where $0 < x < L$ and L is the length of the cilium. The point where $x = 0$ is the open (proximal) end of the cilium, and $x = L$ corresponds to the closed (distal) end. We assume that time t is in the range of several seconds.

The membrane potential satisfies

$$\frac{1}{r_a}\frac{\partial^2 v}{\partial x^2} = -J \tag{1}$$

where r_a is ciliary intracellular axial resistance and J is the transmembrane current flow through the Ca^{2+}-gated Cl^- channels. We have assumed the capacitance term is negligible as well as the background conductance (leak current). The Cl^- current is given by

$$J(x,t) = g_{Cl}\rho(x)F(c(x,t))v(x,t)$$

where g_{Cl} is the single Ca^{2+}-gated Cl^- channel conductance and $\rho = \rho(x)$ is the distribution of the Ca^{2+}-gated Cl^- channels along the length of the cilium. The Hill function

$$F(c) = \frac{c^n}{c^n + K_{1/2}^n} \tag{2}$$

represents Ca^{2+} molecules binding and activating the Ca^{2+}-gated Cl^- channels [47]. Here n is the Hill constant and $K_{1/2}$ is the half-maximal concentration. To complete the description of the membrane potential problem we append the boundary conditions below

$$v(0,t) = v_{Bulk} \quad \text{and} \quad \frac{\partial v}{\partial x}(L,t) = 0. \tag{3}$$

Here v_{Bulk} is the voltage at which the membrane potential is clamped at the open end.

The behavior of the cytoplasmic calcium and buffer complex $b(x,t) = [CaB]$ can be modeled by the following initial/boundary value problems [48]

$$\frac{\partial c}{\partial t} = D_{Ca}\frac{\partial^2 c}{\partial x^2} - F_B(c) + (k_- b - k_+ c(B_T - b)), \tag{4}$$

$$\frac{\partial b}{\partial t} = D_B\frac{\partial^2 b}{\partial x^2} - (k_- b - k_+ c(B_T - b)), \tag{5}$$

and

$$F_B(c) = \alpha B_S \rho \frac{\partial}{\partial t}\left(F(c)\right)$$

where k_+ and k_- are the rate constants for uptake and release of Ca^{2+} from the buffer. Here D_{Ca} and D_B are the diffusivities for the Ca^{2+} and buffer, respectively. We neglect any Ca^{2+} pumps, since no ATP is supplied. B_T is the total concentration of buffer (complex plus free buffer). We neglect calmodulin, which has multiple Ca^{2+} binding sites associated with the CNG channels [49]. The model is not very sensitive to the number of Ca^{2+} binding sites (see the Results section of the main paper).

Assuming that the rapid buffer approximation (RBA) is appropriate (buffer reaction is faster than the other processes involving calcium) we set $K_B = k_-/k_+$ then combine (4) and (5) to obtain [48,50]

$$\frac{\partial c}{\partial t} = \frac{1}{1+\theta}\left[\frac{\partial^2}{\partial x^2}\left(D_{Ca}c + D_B B_T \frac{c}{K_B + c}\right) - F_B(c)\right] \tag{6}$$

where

$$b = \frac{B_T c}{K_B + c} \quad \text{and} \quad \theta = \frac{B_T K_B}{(K_B + c)^2}.$$

This model for the evolution of calcium is complete after adding the boundary conditions

$$c(0,t) = c_{Bulk} \;\text{ and }\; \frac{\partial c}{\partial x}(L,t) = 0 \tag{7}$$

and initial condition

$$c(x,0) = 0. \tag{8}$$

We have represented the fact that there is a free calcium concentration c_{Bulk} outside of the pipette (at the open end of the cilium) and initially the concentration inside is so small that we take it to be zero. We are especially interested in the behavior of the current,

$$I(t) = \int_0^L J(x,t)\, dx,$$

which is experimentally measurable.

Our belief that the RBA is appropriate arises from a nondimensionalization of (4) using $K_{1/2}$ for c and B_T for b. This reveals that the buffer reaction term is much larger than the other terms that model the diffusion and binding of Ca^{2+}. (In fact, the factor is around 10^6). So the reaction term can be solved independently to determine b in terms of c and then substituted into the new equation obtained by adding (4) to (5) leading to (6).

Continuing to follow Keener & Sneyd [48], we can reformulate our model by defining

$$w = \phi^{-1}(c) := D_{Ca}c + D_B B_T \frac{c}{K_B + c}$$

where ϕ^{-1} is a one-to-one function which we can invert by solving an intermediate quadratic equation in c to obtain

$$c = \frac{-(D_{Ca}K_B - w + D_B B_T) + \sqrt{(D_{Ca}K_B - w + D_B B_T)^2 + 4D_{Ca}K_B w}}{2D_{Ca}}.$$

Since

$$\frac{\partial w}{\partial t} = \left(\frac{d}{dc}\phi^{-1}(c)\right)\frac{\partial c}{\partial t} = (D_{Ca} + D_B\theta)\frac{\partial c}{\partial t}$$

we have

$$\frac{\partial w}{\partial t} = \frac{D_{Ca} + D_B\theta}{1+\theta}\left[\frac{\partial^2 w}{\partial x^2} - F_B(c)\right]$$

or

$$\frac{\partial w}{\partial t} = \frac{D_{Ca} + D_B\theta}{1 + \theta + \alpha B_S \rho F'(\phi(w))}\left[\frac{\partial^2 w}{\partial x^2}\right]. \tag{9}$$

Again we append boundary conditions

$$w(0,t) = \phi^{-1}(c_{Bulk}) \text{ and } \frac{\partial w}{\partial x}(L,t) = 0 \tag{10}$$

and initial condition

$$w(x,0) = 0. \tag{11}$$

Remark: In situations where $2c/K_B << 1$, it can be shown that equation (6) is given approximately by

$$\frac{\partial c}{\partial t} = D_{eff}\frac{\partial^2 c}{\partial x^2} - F_B(c) \qquad \text{and} \qquad D_{eff} = \frac{D_{Ca} + D_B B_T}{K + B_T}$$

[48]. If we further assume that the impact of the binding is small and the cilium is long so we can neglect the boundary condition at the tip (resulting in a half-line problem), then a closed form solution for c can be determined. This reduction is reasonable in the case when the buffer is HEDTA since, then, $K_B = 14.8\ \mu$M.

Inverse Solver

Previous studies [9,19,20] suggest that tall, narrow Gaussian channel distributions provide current evolution profiles that fit the experimental current data well (see also Fig. 2 of Results). We thus search for x_0, ρ_0, and δ_w so that the current $I(t)$ resulting from

$$\rho(x) = \rho_0 \exp(-\left(\frac{x - x_0}{\delta_w}\right)^2)$$

in our model is a good match to the measured current data for a given specific cilium. Here ρ_0 is the peak height, x_0 is the location of that peak and δ_w is a width. Note the following approximate equality that holds if the Gaussian is truly narrow with respect to the length of the cilium L and not centered close to the ends

$$T_{Cl} = \int_0^L \rho(x)\ dx \cong \int_{-\infty}^{\infty} \rho(x)\ dx = \rho_0\delta_w\sqrt{\pi} \tag{12}$$

where T_{Cl} is the total number of channels. So the matching involves choosing x_0, ρ_0, and δ_w so that

$$S(x_0, \rho_0, \delta_w) = \frac{1}{N_{Data}}\sum_{i=1}^{N_{Data}} (I(t_i) - I_i^{Data})^2$$

is minimized where $(t_1, I_1^{Data}), \ldots, (t_{N_{Data}}, I_{N_{Data}}^{Data})$ are the time and current data points arising from the experiments (e.g. those plotted in Fig. S4 below). Our search procedure uses asymptotic formulas from Badamdorj et al. [20] in concert with a dichotomous search algorithm to provide a good guess for x_0 followed by the MATLAB *fminsearch* code that uses the Nelder-Mead algorithm to find the three points x_0, ρ_0, and δ_w.

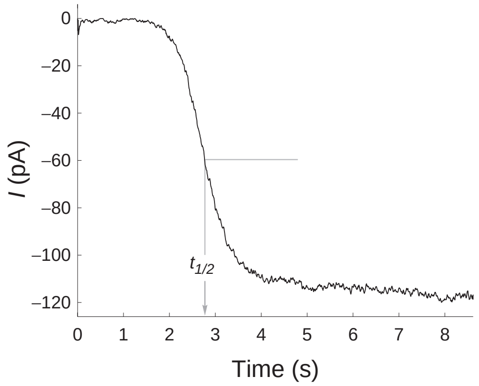

Figure S4. Time course of the experimental current. Current is shown from time 0, when the cilium was immersed in the buffer/Ca^{2+} bath. Here $t_{1/2}$ is the time at which the current reaches half its maximum depth. In the example shown, $t_{1/2} = 2.8\ s$ and the maximum current depth $I_{Cl} = -120$ pA.

We describe the dichotomous search part of the overall algorithm more precisely. The reduced problem studied in Badamdorj et al. [20] indicated that if $t_{1/2}$ is the time the current profile has reached half its peak height I_{Cl} (see Fig. S4) and $D_{Avg} = \frac{1}{2}(D_{Ca} + D_B)$ then

$$x_0 \cong \frac{\sqrt{\pi D_{Avg} t_{1/2}}}{1 + D_B B_T (D_{Ca} c_{Bulk})^{-1}} \qquad \text{and} \qquad T_{Cl} \cong \frac{I_{Cl}}{g_{Cl}(v_{Bulk} - r_a I_{Cl} x_0)}. \tag{13}$$

So we search for a minimizing argument x_0 of

$$F_M(x_0) = S(x_0, \frac{I_{Cl}}{g_{Cl}\sqrt{\pi}\delta_w (v_{Bulk} - r_a I_{Cl} x_0)}, \delta_w)$$

where we used (12) and the second formula in (13) for ρ_0 in the sum of squares error formula S.

Dichotomous Search Algorithm:

Choose $[a_D, b_D]$, ϵ_D, and i_{Max}.
Set $i = 0$ and $\delta_w = 1$.
While $i < i_{Max}$.
    $i = i + 1$.
    $c_D = (a_D + b_D)/2$.
    $c_D^- = c_D - \epsilon_D$ and $c_D^+ = c_D + \epsilon_D$.
    If $F_M(c_D^-) > F_M(c_D^+)$ then $a_D = c_D$ else $b_D = c_D$.
end while loop.

The dichotomous search code assumes the function F_M is unimodal. It is a bracketing approach like the bisection method for rootfinding. Successive subintervals are determined by bisection and calculation on the half subinterval in which the minimum resides. A distinguishability coefficient ϵ_D is used for this ($0 < \epsilon_D << 1$). A bracketing interval $[a_D, b_D] \subset [0, L]$ is initially found from the guess for x_0 in (13). We have generally chosen $\delta_w = 1$ in this search. Of course in the MATLAB search we allow all three parameters (x_0, ρ_0, δ_w) to vary.

Usually, only a moderate number of iterations are done, since there is the second Nelder-Mead search as well. This *fminsearch* or Nelder-Mead search starts with a guess using the x_0 from the dichotomous search, a value for ρ_0 from (12) and (13), and $\delta_w = 1$.

Example

Here we display the graphs from a sample run of our code which searches for an optimum (x_0, ρ_0, δ_w) set of parameters. The results in Fig. S5 are for a 25-μm cilium.

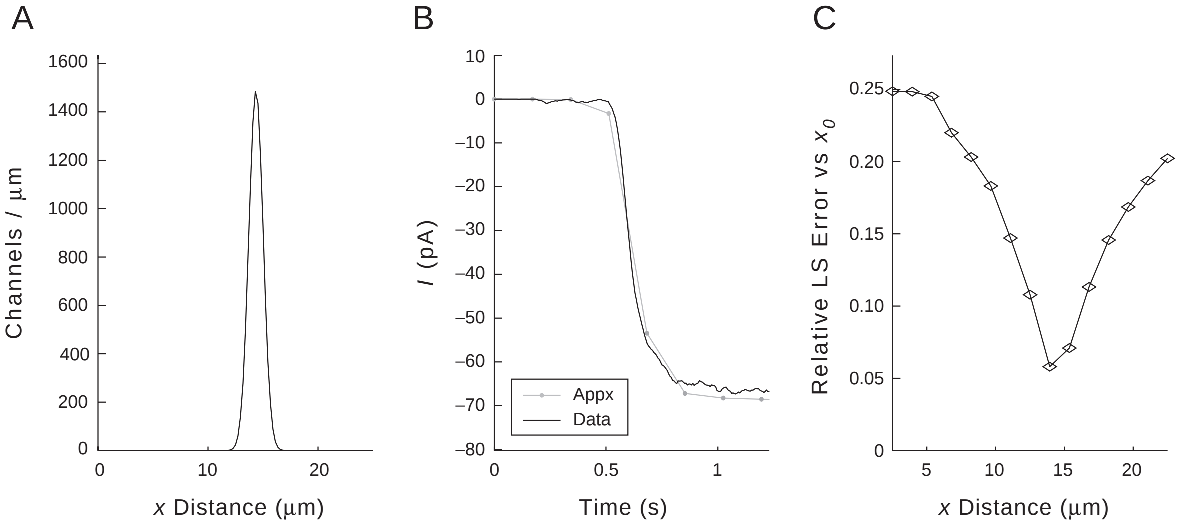

Figure S5. Results from an example run with the inverse solver. A, plot of the channel density function ρ found by the full inverse solver with 8 dichotomous and 8 Nelder-Mead iterations. B, current from experimental (black) and forward solver with ρ (gray). C, Functional F_M from the dichotomous search displaying that it is unimodal.

The inverse solver was used and produced an answer with a relative least squares error where $E_2 = 0.024$ with

$$E_2 = \frac{\sqrt{S(x_0, \rho_0, \delta_w)}}{\sqrt{\frac{1}{N_{Data}} \sum_{i=1}^{N_{Data}} (I_i^{Data})^2}}.$$

Fig. S5A is a plot of the channel distribution function ρ with $x_0 = 14.4$ μm, $\delta_w = 0.917$, and $\rho_0 = 1489$ channels. Here the channel density was 96.8 channels/μm and total number of channels $T_{Cl} = 2420$. In Fig. S5B are shown the current data resulting from a forward solution using ρ (gray) as well as the experimental current data (black). In Fig. S5C there is a plot of $F_M(x)$ vs x revealing that it is unimodal in this example.

References cited that do not appear in the main article

46. Badamdorj D (2006) Mathematical modeling of the Cl(Ca) ion channels in frog olfactory cilia. Ph.D. thesis, University of Cincinnati, Cincinnati, OH.

47. Lindemann B (2001) Predicted profiles of ion concentrations in olfactory cilia in the steady state. Biophys J 80: 1712-1721.

48. Keener J, Sneyd J (1998) Mathematical Physiology. New York: Springer-Verlag.

49. Bradley J, Bönigk W, Yau K-W, Frings S (2004) Calmodulin permanently associates with rat olfactory CNG channels under native conditions. Nature Neurosci 7: 705-710.

50. Smith GD (2001) Modeling local and global calcium signals using reaction-diffusion equations. In: De Schutter E, editor. Computational Neuroscience / Realistic Modeling for Experimentalists. CRC Press, Boca Raton, FL: CRC Press. pp. 49-86.

Table S2: Model Data – Parameters and Definitions

Label	Name	Value	References
L	Length of cilium	$18\ \mu\text{m} \le L \le 78\ \mu\text{m}$	
R_i	Ciliary intracellular resistivity	$9.2 \times 10^{-4}\ \mu\text{m nS}^{-1}$	[116 mM LiCl]
r_c	Ciliary radius	$0.14\ \mu\text{m}$	16
r_a	Ciliary intracellular axial resistance ($R_i/\pi r_c^2$)	$1.5 \times 10^{-2}\ (\text{nS}\ \mu\text{m})^{-1}$	
g_{Cl}	Single Ca^{2+}-gated Cl^- channel conductance	$(8.0 \pm 0.9) \times 10^{-4}$ nS channel^{-1}	27
n	Hill constant	2.00 ± 0.094	21
$K_{1/2}$	Ca^{2+}-gated Cl^- channel activation constant	$4.8 \pm 0.29\ \mu\text{M}$	21
v_{Bulk}	Voltage-clamp potential	$-50, -40,$ or $+40$ mV	
D_{Ca}	Ca^{2+}diffusion coefficient	$300\ \mu\text{m}^2\ \text{s}^{-1}$	48
k^+, k^-	Buffer reaction rate constants	$600\ \mu\text{M s}^{-1}$, $100\ \text{s}^{-1}$	50
B_T	Total concentration of buffer	200, 600, or 2000 μM	
c_{Bulk}	Free calcium concentration in bulk	7, 20, or 300 μM	
α	Conversion factor	$2.7 \times 10^{-2}\ \mu\text{M}\ \mu\text{m molecule}^{-1}$	19
B_S	Number of binding sites	1 molecule channel^{-1}	
K_B	BAPTA reaction rate	$0.157 \pm 0.003\ \mu\text{M}$	see Methods
K_B	DBB reaction rate	$1.24 \pm 0.05\ \mu\text{M}$	see Methods
K_B	HEDTA reaction rate	$14.8 \pm 0.4\ \mu\text{M}$	see Methods
D_B	Buffer diffusion coefficient	$95\ \mu\text{m}^2\ \text{s}^{-1}$	50

Notes:

- Where uncertainties are shown they represent SEM. Uncertainties given for n and $K_{1/2}$ are unpublished values from the data in Kleene & Gesteland [21].
- For the conversion factor above, $\alpha = 1/(N_A A)$ where A is the cross-sectional area of the cilium and N_A is Avogadro's number.
- The reaction rates are apparent dissociation constants between Ca^{2+} and the buffer, i.e. the inverses of the apparent association constants determined as described in the Methods section of the main paper.
